# A Comprehensive Review of Sensors and Instrumentation Methods in Devices for Musical Expression

**DOI:** 10.3390/s140813556

**Published:** 2014-07-25

**Authors:** Carolina Brum Medeiros, Marcelo M. Wanderley

**Affiliations:** Input Devices and Music Interaction Laboratory (IDMIL), Centre for Interdisciplinary Research in Music Media and Technology (CIRMMT), McGill University, 555 Sherbrooke St West, Montreal, QC H3A 1E3, Canada; E-Mail: marcelo.wanderley@mcgill.ca

**Keywords:** sensor fusion, strain gages, accelerometers, FSR, digital musical instruments, Do-It-Yourself, NIME conference

## Abstract

Digital Musical Instruments (DMIs) are musical instruments typically composed of a control surface where user interaction is measured by sensors whose values are mapped to sound synthesis algorithms. These instruments have gained interest among skilled musicians and performers in the last decades leading to artistic practices including musical performance, interactive installations and dance. The creation of DMIs typically involves several areas, among them: arts, design and engineering. The balance between these areas is an essential task in DMI design so that the resulting instruments are aesthetically appealing, robust, and allow responsive, accurate and repeatable sensing. In this paper, we review the use of sensors in the DMI community as manifested in the proceedings of the International Conference on New Interfaces for Musical Expression (NIME 2009–2013). Focusing on the sensor technologies and signal conditioning techniques used by the NIME community. Although it has been claimed that specifications for artistic tools are harder than those for military applications, this study raises a paradox showing that in most of the cases, DMIs are based on a few basic sensors types and unsophisticated engineering solutions, not taking advantage of more advanced sensing, instrumentation and signal processing techniques that could dramatically improve their response. We aim to raise awareness of limitations of any engineering solution and to assert the benefits of advanced electronics instrumentation design in DMIs. For this, we propose the use of specialized sensors such as strain gages, advanced conditioning circuits and signal processing tools such as *sensor fusion*. We believe that careful electronic instrumentation design may lead to more responsive instruments.

## 1. Introduction

The use of sensors and associated signal conditioning to measure physical quantities involves the fields of metrology and electronic instrumentation. Metrology is the science of measurement and its application. The metrology science has its own terminological dictionary that determines the “basic principles governing quantities and units”: the International Vocabulary of Basic and General Terms in Metrology (VIM) [1]. The VIM, along with the GUM (Guide to the Expression of Uncertainty in Measurement), define uncertainty and errors involved in measurements. Electronic instrumentation is the measurement chain of an electronic measuring system, resulting in an analog or digital electrical output quantity. An electronic instrumentation typically includes all signal conditioning techniques on the path of a sensor signal towards an output electrical value. A sensor is considered the “element of a measuring system that is directly affected by a phenomenon carrying a quantity to be measured” [1]. Signal conditioning techniques include procedures and circuits devoted to adjust, amplify, filter, select and transduce signals. Instrumentation of any sensor signal implies errors and uncertainties. Peter K. Stein states that a critical question concerning measurements is: “Could these data have been acquired by that measurement system without distortion, contamination and without affecting the process being observed” [2]. In order to answer this question, he developed the “Unified Approach to the Engineering of Measurement Systems for Test and Evaluation”. This Approach summarizes techniques to test and evaluate measuring systems [2,3]. However, advanced electronic instrumentation is not sufficient to deal with the limited capability of measuring systems. Techniques such as calibration, regression, physical modeling, classification and sensor fusion are helpful tools to enhance measurements. A reminder about the importance of evaluating measured data is given by Stein: “Bad data look just as believable as Good Data! We ask a Measurement System for the Facts, not for its Opinion!”[2].

The development of electronic instrumentation for artistic applications is even farther from trivial. Buxton states that “in the grand scheme of things, there are three levels of design: standard spec, military spec and artist spec. Most significantly, I learned that the third, artist spec, was the hardest (and most important). If you could nail it, then everything else was easy” [4]. We believe that this applies to the design of musical tools, such as musical instruments. Indeed, expert musicians develop, over several years, very high motor control skills to perform their acoustic instruments. For this, they rely on generally stable, robust and responsive acoustic musical instruments that result from centuries of *lutherie* knowledge. Stable instruments are a necessary but not sufficient requirement for musical expression. As Dobrian and Koppelman state: “sophisticated musical expression requires not only a good control interface but also virtuosic mastery of the instrument it controls” [5]. In the last few decades, Digital Musical Instruments (DMIs) gained popularity among a large population. Several performers use DMIs in their practice and some of them have developed very high motor control skills to perform these instruments. The design of a DMI offers few physical constrains and it is a highly creative endeavor involving a variety of knowledge fields such as art, design, human-factors and engineering. The balance between these areas is a delicate issue. Cook states that “Musical interface construction proceeds as more art than science, and possibly this is the only ways that it can be done” [6]. We would rather advocate for a balanced approach between art and science. Highly artistic instruments with poor engineering solutions might hinder musical expression as the instruments might not satisfy skilled performers' needs.

Dobrian and Koppelman wrote their 2006 NIME paper in order to “draw attention to the question of whether musical expression in performance is being adequately addressed in much current research on real-time computer music interfaces” [5]. With this paper, we have similar goals concerning the sensing design. We are convinced that by applying advanced engineering techniques, one can achieve the hard requirements expressed above and create responsive instruments that marry highly artistic design with state-of-the-art engineering solutions.

As most of DMIs are meant for real-time performance, instrumentation techniques providing stable, robust, accurate, reproducible and fast response are essential. Despite these demanding design requirements, a large number of DMIs are currently developed in a Do-It-Yourself (DIY) manner using techniques that often result in unsophisticated engineering solutions that prioritize easily available sensors that are simple to assemble and require uncomplicated signal conditioning circuits [7,8]. Furthermore, the main academic event related to DMIs, the International Conference on New Interfaces for Musical Expression (NIME), presents a similar trend. A review of the first eight years this conference showed that the majority of NIME DMIs are also based on a few habitual sensor technologies [9].

In this work, we focus our attention on the engineering design of DMIs, particularly the choice of electronic instrumentation strategies. We present an overview of the electronic instrumentation strategies used in 266 papers by the NIME community from 2009 to 2013. We report the most commonly used sensors as well as their concomitant use with the same application. These results confirm the previous observations by Marshall *et al.* [10]. Disregarded in the previous survey, in the recent years, we identify the use of portable consumer electronics devices such as cell phones, tables and game controllers trending up. Following this trend, we verify the usage of sensors embedded in these devices. In addition, we survey the motion capture methods used by the community. We further identify other significant trend of measuring force-related quantities, using either accelerometers or Force Sensing Resistors (FSRs). Several of these force measurements do not follow application guidelines and sometimes their data analysis do not relate to any physical meaning. Due to that, we conclude that there is room for improvement in the instrumentation design of DMIs and here we introduce three main directions for improvement: the use of *specialized sensors, advanced instrumentation techniques* and *signal processing tools* such as *sensor fusion*.

We introduce strain gages as an option for force measurement. Although these sensors present complex application and conditioning circuits, they provide high reproducibility, linearity and monotonicity. Our suggestion for advanced electronic techniques focuses on ordinary sensors and include circuits for gain and offset control, amplification, common-mode rejection and stable switching for discrete state measurements. Furthermore, we show that the use of sensor fusion techniques can lead to results whose errors are smaller than the error of each individual data source. In order to offer the reader a perspective of alternative ways to develop electronic instrumentation and sensor signal processing for DMIs, we comment the development of a few NIME papers, offering progressive solutions using one of the three solutions: *specialized sensors, advanced instrumentation techniques* and *signal processing tools*.

### Previous Work

**Figure 1.**
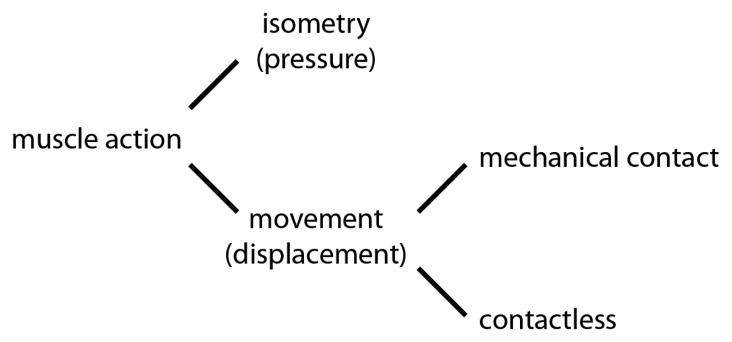
Taxonomy used by Bongers [11]. Movements starts with human muscle action and can be further distinguished into isometric or movement [11]. Reproduced with permission.

Several studies are dedicated to review physical interfaces for musical expression. Some works borrow concepts of HCI (Human Computer Interaction) to define a physical interface features and evaluation [11,12]. Bongers' description of sensor types and uses is based on an association of those with *human muscle action* [11]. His classes of muscle action are reproduced in Figure 1. For each one of these classes, he cites several sensors. It is interesting to note that the author was able to classify a huge variety of sensor types using the variables *pressure and displacement*. These two variable are highly correlated to the two biomechanical factors on human motion: *kinetics* and *kinematics*. Bongers' paper is part of an electronic publication of articles discussing *Trends in Gestural Control of Music* [13]. More recent publication in the physical interfaces for musical expression reviews gesture definitions in music and simple sensing technologies [14].

Although several studies introduce different techniques for gesture acquisition and analysis using motion capture and/or sensors [15–19], not many studies are devoted to the interface between DMI design and sensor technology [9–11]. Existing surveys of sensor use in DMIs show that only a restricted number of common sensors are used in most applications [9,10]. There are nevertheless several sensors, conditioning circuits and processing techniques that, due to their complexity and processing times, remain unknown among DMI designers [20,21]. It is worth noting that concerning conditioning circuits, it is hard to find a unique solution that fits a variety of sensors, measurement ranges and applications. This might explain why just a few engineering books describe conditioning circuits for each sensor type [20].

The literature on sensing technology is vast and not restricted to academic publications. Manufacturers of sensor and signal conditioning ICs (Integrated Circuits) have a huge contribution with their *datasheet* and *application guideline* publications [22–25]. For testing and evaluation of measuring systems, the GUM Guide and the Unified Approach of Stein are essential references [3,26]. Sensor fusion techniques are discussed by several works [27–32], but only a few of them focus on sensor fusion specifically for human body applications [33–39]. Indeed, the most popular application of fusion algorithms such as Kalman filters is devoted to navigation and tracking estimates using inertial and magnetic sensors, including those for human body applications [40,41]. A few studies are dedicated to sensor fusion using other sensor types and sensor fusion with uncertain process model [42,43]. A handful of publications describe in detail instrumentation and/or processing techniques such as calibration for inertial and magnetic sensor data [44–46].

Several NIME papers are dedicated to survey relevant sensing/processing techniques for the community: motion capture tools [47–49], machine learning [50] and the use of mobile devices [51]. Some NIME-related studies are based on advanced engineering techniques and sensor technologies, both published in NIME and elsewhere. For instance, the use of specialized sensors—which require advanced conditioning circuit techniques—can be found in a few papers [42,52–56]. Besides that, sensor fusion approaches are cited in some studies, although their implementation is not reported [57].

## 2. Review of Sensor Use

First, we investigate the use of sensors in the DMI community from the last five years as manifested in the NIME conference proceedings (NIME 2009–2013) yielding a critical evaluation of sensor application and data interpretation. Table 1 presents the sensor use summary, compared with the dataset of previous study [10]. Some remarks about these categories are presented below in Table 1 and numbered according to the superscripted and numbered marks. We aimed to classify sensor use in DMIs in terms of the type of sensors and quantity to be measured. However, due to the varying clarity of NIME manuscripts, it is not always possible to distinguish and classify the DMIs according to the quantities being measured and the sensors used. Often, these two concepts are blurred somewhat. For example, some authors say that “we have used a touch sensor”. In this case, *touch* is the quantity to be quantified, whereas a *touch sensor* can be a capacitive or resistive sensor. Despite the effort towards classifying sensors and quantities in DMIs, sometimes it was not possible to determine the technology used nor the physical quantity to be measured. These cases are categorized as non-definable.

Sensors that were similarly classified in both studies have their average incidence per area presented in Table 2. A quick glance at Tables 1 and 2 shows some interesting facts that require further exploration:

- Accelerometers and FSRs are the most used ones, similar to previous findings [10];
- The most popular sensors measure force indirectly, e.g., FSRs (Force Sensing Resistors) and accelerometers.

Table 3 shows the non-exclusive classification of occurrences per class. The non-exclusive occurrence means that an NIME application can be classified in multiple classes according to the resources (sensors/devices/equipments) it uses. The criteria for the classes are described as follows:

**Analog sensors:** sensors that output a continuous electrical signal [20];
**Digital sensors:** sensors that output a discrete electrical value: step or state [20]. They might be embedded in a device or not;
**Consumer electronics:** portable devices primarily commercialized as devices for everyday use, mostly for entertainment or communication. In this context, it includes portable music players, cell phones, Wii^©^, Kinect^©^ and tablets. Some NIME applications use the device's own functions modified for a particular function, whereas others use data from the device's embedded sensors;
**Motion capture:** refers to Kinect^©^, infrared camera-based systems such as Qualisys^©^, commercially available bodysuit sensor nodes such as the Xsens^©^ and electromagnetic sensors nodes such as Polhemus^©^. Two remarks concerning this classification must be made. The first one refers to labeling the Kinect^©^ as both consumer electronics and a motion capture tool. The second one refers to the distinction made between body sensor systems, such as the commercially available Xsens^©^, and dedicated solutions using sensors—classified as *digital sensors*. Both solutions are usually based on the same sensors: accelerometers, gyroscopes and magnetometers, however the first is presented like a black-box system whereas the second is a compound of sensors placed together and configured throughout. Accelerometers, gyroscopes and magnetometers are called MARG — (Magnetic, Angular Rate, and Gravity) — sensors.

Additional survey analyses on sensor use in DMIs follow. These analyses show interesting trends and sensor co-occurrences of the NIME designs.

### 2.1. Interesting Trends

**Figure 2.**
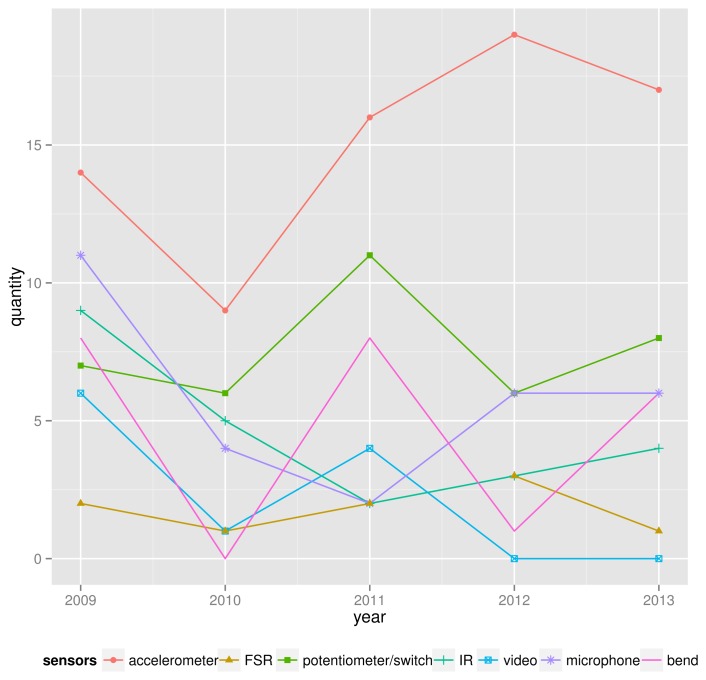
Trends for some sensors within the interval 2009–2013.

According to Figure 2, some interesting trends are noticeable:

- Accelerometers (embedded or not) were the most popular sensor across the years;
- FSR use is stable over the years;
- Potentiometers and switches are substantially used;
- The latest years (2012–2013) show that video has not been used recently;
- IR, microphone and bend sensors do not present a clear trend.

### 2.2. Use of Portable Consumer Electronics

**Figure 3.**
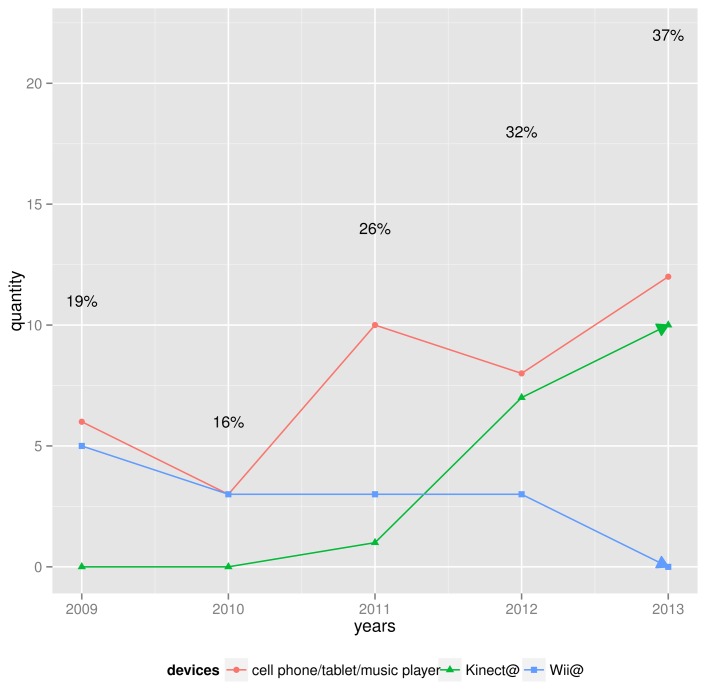
Consumer electronics use within the interval 2009–2013. The percentage numbers reflect the percent of portable devices use compared with the total number of measuring techniques reviewed per year.

The use of consumer electronics was not cited in the previous DMI survey [10]. This suggests that their use has only become substantial recently. Figure 3 shows the use of these devices over the past five years. The percentage numbers reflect the percent of portable devices use as compared with the total number of measuring techniques reviewed per year. Note that since the use dropped in 2010, the use of portable consumer electronic devices in DMIs has monotonically increased, except for the Wii^©^.

#### 2.2.1. Embedded Use of MARG (Magnetic, Angular Rate, and Gravity) Sensors

MARG sensors comprise accelerometer, gyroscope and magnetometer sensors. Table 4 expresses the embedded use of these sensors in portable consumer electronic devices, through the study of their co-occurrence. The numbers show that most of the cell phone, music players and tablet applications make use of their embedded accelerometer. The embedded use of gyroscopes and magnetometers has fewer occurrences as their availability on portable consumer electronics is more recent.

### 2.3. Clusters

**Figure 4.**
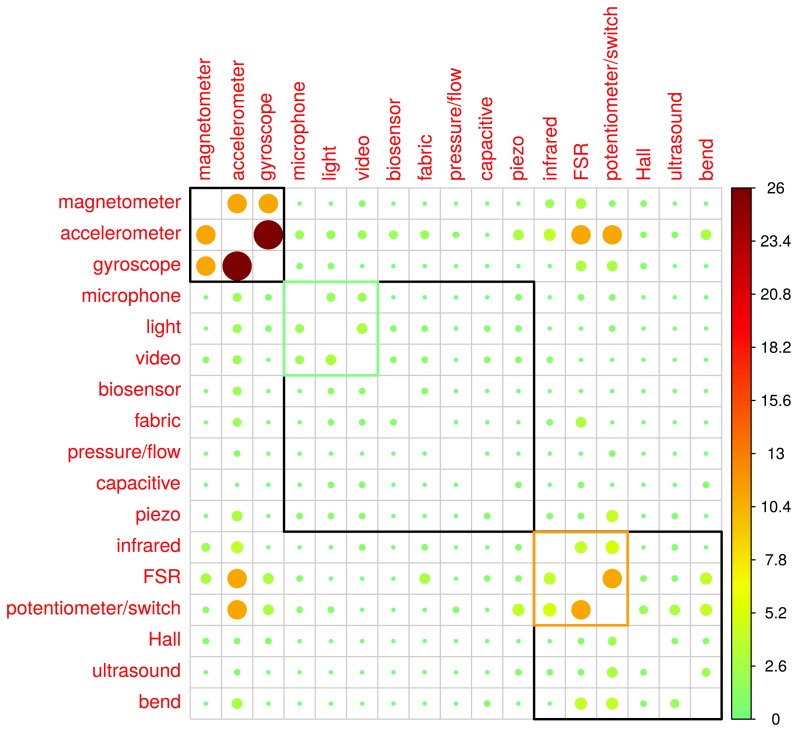
Co-occurrence matrix map plus Ward hierarchical clustering for 3 clusters. As the co-occurrences of an aspect with itself were shown in Table 1, the main diagonal is blank for clarity. Upper and lower matrices report the same information. The green and orange squares present the 4 clusters solution.

In this section, the co-occurrence of two sensors is analyzed through their adjacency matrix. This matrix quantifies the concomitant use of two sensors within the same application. Mapping the co-occurrence matrix results in Figure 4. A Ward hierarchical cluster algorithm was run for 3, 4 and 5 clusters [59]. The results for the choice of 3 clusters are highlighted in Figure 4, which also depicts the results for 4 clusters. This algorithm uses the minimum variance within clusters as the criterion. Therefore, the clusters are formed for sensors that have similar co-occurrence values among each other. Given an approach with 3 clusters, the first cluster includes inertial and magnetic sensing (MARG sensing). These sensors along with well-designed sensor fusion techniques can provide accurate orientation data. As such, they are often deployed for navigation and more recently for human motion analysis. This will be discussed in Section 3.1. A second cluster is formed by infrared, FSR, potentiometers/switches, Hall effect, ultrasound and bend sensors. A common feature among all these sensors is that they can be easily assembled and they have a relatively low cost. They are cited among the most commonly used sensors in musical applications [10,14]. Also, they are ubiquitous in forums and tutorials on sensors and microcontrollers [60–62]. The remaining cluster groups the sensors that tend to be used alone or with a few other types of sensing technologies; these are: microphone, light, video, biosensor, fabric, pressure/flow, capacitive and piezoelectric disc. For the 4 clusters solution, the clusters are: MARG sensors; infrared, FSR and potentiometer/switch, microphone, light and video; and the remaining.

**Figure 5.**
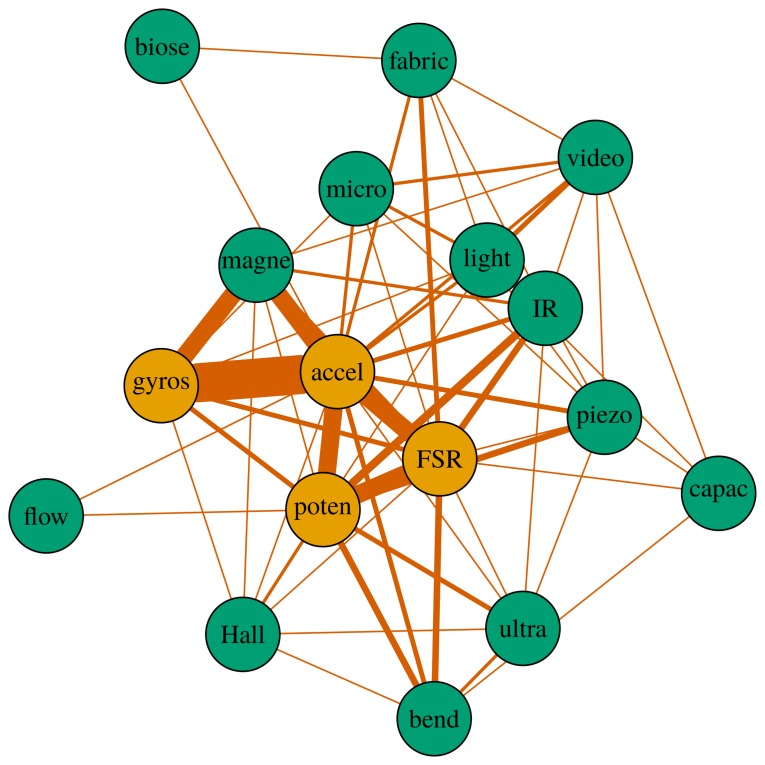
Co-occurrence of sensors: line thickness is proportional to the number of instances the connected sensors were used in the same application. Sensor names were shortened as: *biose*, biosensor; *micro*, microphone; *flow*, pressure/flow; *accel*, accelerometer; *gyros*, gyroscope; *magne*, magnetometer; *poten*, potentiometer/switch; *capac*, capacitive; *ultra*, ultrasound. Sensors with the highest *degree* appear in orange.

Simple econometrics for social network analysis was deployed in order to better describe the concomitant use of sensors in DMIs. In this analogy, sensors are seen as users connected through a network of DMIs. In addition, the cited clusters can be seen as communities. The graph in Figure 5 is designed using an algorithm for undirected graphs called Kamada Kawai. This algorithm defines the ideal distance between the elements in order to provide a total balance of the graph and a small amount of edge crossings [63].

The image clearly shows the two strong clusters: one formed by the MARG sensors and another by resistive-based sensors (potentiometer/switch, FSR). It is noticeable that the accelerometer is placed in the center of the network. This comes from the fact that this sensor presents the highest *degree* of the network. The *degree* of a node expresses the number of connections that an object has [64]. The accelerometer is concomitantly used with 17 other sensing types. Potentiometers/switches and FSR have the second highest degree. Similar analysis was performed including *consumer electronics* and *motion capture* tools. The results—not pictured—show the following:

**Motion capture:** the incidence of motion capture tools coincides with a limited variety of other sensing techniques. Motion capture tools are only used in conjunction with other motion analysis sensing techniques—MARG sensors and Wii^©^—and video. This finding suggests that designers aiming to perform motion analysis do not consider motion capture tools as a unique and sufficient method to do so. This discussion will be presented in Section 3;
**Wii^©^:** the measuring capabilities of this device used in DMIs are its infrared camera and its accelerometer. Seven out of fourteen times this device was used, its embedded accelerometer data was used;
**Kinect^©^:** of the 18 times the Kinect^©^ was used, only once was another sensing technique—accelerometer—used.

## 3. Motion Analysis Sensing

DMIs are controlled by human input, thus their signals are difficult to predict or to classify. Most of human input to DMIs is essentially motion, which comprises kinematic and kinetic features. Kinematics is the study of motion and its variables, whereas kinetics is the study of internal and external forces and their momentum [15]. Regarding the sensor use, accelerometers, gyroscopes and FSRs can provide kinetic data, whereas Kinect^©^, infrared- and electromagnetic-based tools can provide kinematic data.

In this section we discuss MARG sensors and motion capture tools for biomechanical analysis of human motion. The appeal of MARG sensing had a large impact on most recent NIME papers. This can be observed in the numerous works willing to make the body itself as a DMI controller, instead of using an object for sensing human motion [14,65–68]. Several NIME papers report the use of more than one motion analysis tool in the same application, some including kinetic and kinematic methods. This suggests the implementation of sensor fusion techniques to take advantage of the best features of each sensing technique.

### 3.1. MARG Sensors

**Figure 6.**
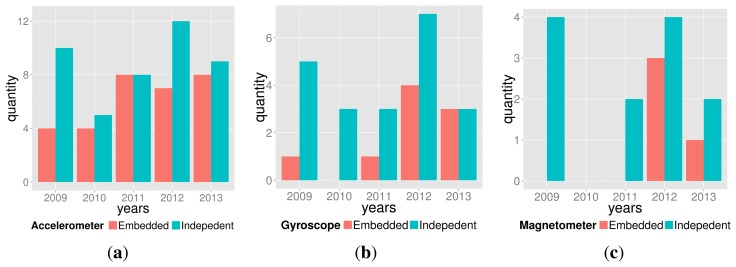
Embedded and independent use of MARG sensors. (**a**) accelerometer; (**b**) gyroscope; (**c**) magnetometer.

First, a remark must be done about terminology. Often, DIY and NIME literature use the term IMU—Inertial Measurement Unit—to refer to concomitant use of accelerometer, gyroscope and magnetometer [49]. Accelerometer and gyroscopes are indeed inertial sensors, but magnetometers are not. The scientifically accurate terms are *MARG sensors* or *AHRS system*. MARG—Magnetic, Angular Rate, and Gravity—sensors refer to the concomitant use of the three cited sensors, whereas AHRS—Attitude and Heading Reference System—refers to a *system* that provides orientation data given the availability of MARG sensors and sensor fusion algorithms. These sensors can either be part of a consumer electronic device—called *embedded*—or part of a dedicated design—called *independent*. Figure 6 depicts the embedded and independent use of MARG sensors.

A brief overview of IMU and MARG sensors use in NIME follows:

- IMU: 24 out of 75 projects using accelerometers also use gyroscopes;
- MARG: 11 out of 75 projects using accelerometers also use both gyroscopes and magnetometers.

The simultaneous use of accelerometer, gyroscope and magnetometer allows the application of sensor fusion filters to provide orientation estimate. Few works (3 out of 266) implement complementary filtering, which has application and processing requirements that are more suitable for embedded applications than Kalman filtering [57]. The implementation of Kalman filters combined with system physical modeling result in estimates with considerable improvement in their error profile. Accelerometer data analysis will be further analyzed in Section 4. Sensor fusion will be discussed in Section 5.2.

### 3.2. Motion Capture

The motion capture techniques used by NIME researchers are infrared-based cameras, magnetic-based sensors, Kinect^©^ and commercially available sensor networks. Many researchers have used the Kinect^©^—primarily available as video game accessory—due to its price and simplicity, in comparison with the infrared- and magnetic- based methods [69]. All of these systems present advantages and drawbacks and the choice depends on the resources available, the problem to be solved and the level of accuracy desired [47,48].

## 4. Force Assessment

In this section, we provide a literature review on force assessment and discuss techniques to improve results, highlighting the most common flaws in the use of FSRs and accelerometers.

### 4.1. Accelerometer

This sensor has become increasingly present in portable consumer electronics, due to advances in micro-machinery technologies. This trend has also made them available at relatively low cost for engineers and designers willing to measure acceleration in their custom design. In fact, accelerometers can provide information not only about translational acceleration, but also about force, vibration, shock and tilt [70].

The use of accelerometers for inclination measurement is not linear unless a narrow range of inclination is considered. Another requirement for inclination measurement is that the Root-Sum-Square (RSS) of the three axes must equal one times gravity [71].

Einstein's equivalence principle states that it is impossible to distinguish gravity from acceleration due to motion [72]. For this reason, when determining the sensor orientation, other sources of data and calibration are necessary. Einstein's observation is one of the reasons why the derivation of position from acceleration data is a topic that requires further discussion.

Rotational motions result in an apparent AC (Alternate Current) acceleration, even if there is no translational acceleration. This apparent acceleration is a consequence of the variable projection of gravity on the axes [71].

In recent times, accelerometers have been used together with gyroscopes and magnetometers, allowing for the full description of orientation, through the use of sensor fusion techniques such as the Kalman filter.

#### 4.1.1. Error Sources

Some sources of errors on accelerometers are [73–75]: nonlinear effects in scale factor, cross-axis coupling, measurement bias, vibro-pendulous error (for pendulous design) [76], drift terms, misalignment errors, Vibration Rectification Error (VRE), quantization, thermo-mechanical white noise, ratiometric errors [77] and random noise.

An error model can consider different amounts of error sources [75,78]. According to several authors, the major deterministic error sources are the zero-offset bias and the first order scale factor [79]. In addition to that, it is necessary to stochastically model the random noise. This modeling requires a minimum span of observation time, which depends on the time constant of the process and on the accepted error level.

#### 4.1.2. Calibration

A calibration process must be able to account for deterministic errors such as bias and scale factor errors. In order to eliminate stochastic sources of error, additional information is required. Several calibration protocols can be found in the literature [44,73,79,80]. Here, we focus on describing one of them due to its high measurement range and the familiarity of the authors with the procedure [44].

The studied calibration protocol is based on properties of the centripetal acceleration. There is a centripetal acceleration when an object is rotated at a given radius greater than zero:

(1) $$a=\omega^{2}r$$

where *a* is the acceleration, *ω* is the angular velocity and *r* is the radius. Therefore, an accurate motor delivers stable angular rates that are correlated to calibration points for acceleration [44]. The angular velocity is sustained for some seconds—sufficient time to gather the minimum amount of samples to guarantee calibration coherence. The stability of the rotation radius is given by placing the sensor node in customized 3D fabricated brackets that are firmly attached to the shaft.

One of the big issues is to generate positive and negative centripetal acceleration with positive angular velocity. A positive acceleration is generated when the accelerometer is rotated in such a position that the rotation axis is positively displaced on the orthogonal plane from its center axis. Alternatively, a negative acceleration is generated by a rotation in which the axis is negatively displaced in relation to the orthogonal plane [44].

#### 4.1.3. Common Issues on Accelerometer Use

In this section, we focus on common issues in the NIME literature, concerning the accelerometer's application and data interpretation. A common application of accelerometer data is to estimate beat through the analysis of gestures of conducting, bouncing, percussion and dance [81,82].

There are several accelerometer data processing solutions that can be found in DIY tutorials and NIME papers, most of them for extracting beat information. Some are:

(1) Sum of the value of the axes;
(2) Use of the highest component of the acceleration;
(3) Peak detection;
(4) Norm of the acceleration;
(5) Norm less gravity;
(6) FFT (Fast-Fourier Transform);
(7) Acceleration integration;
(8) Machine learning techniques.

The first solution does not present any physical meaning. The second one does not take into account the orientation of the sensor. Changes in orientation will bleed gravity and motion acceleration throughout the axes. The designer should reject any conclusions when the highest component is not sufficiently greater than gravity. Peak detection might be a coherent but not sufficient technique. The designer may remember that physical interpretation of zero-crossings and peaks on position data, velocity data and acceleration data are different. Only for a simple periodical signal, the acceleration peaks relate frequency-wise to those in the position domain.

Concerning processing cost, the least expensive solutions that have connection with the physical world are the options 4 and 5. The norm of the acceleration represents the absolute value of acceleration—gravity and motion acceleration. The norm subtracted by the absolute value of the gravity acceleration provides the magnitude of the acceleration due to motion.

FFT—possibly along with windowing techniques—and acceleration integration have the most expensive processing cost. The FFT is not a possibility for all types of signals though. The double integration in order to obtain position data leads to errors due to uncertain integration constants. Finally, pattern recognition, feature selection and a variety of other machine learning tools, usually aiming gesture classification, could be used [50,83].

### 4.2. FSR

Most of the FSRs are a polymer thick film device that vary their resistance according to the pressure applied to the active surface. The FSR manufacturer Interlink™ claims FSRs are not load cells or strain gages, although they have similar properties [84]. Besides, Interlink™ claims that FSRs are not suitable for precision measurements: force accuracy ranges from 5% to 25% [84]. According to the analysis of commercially available FSRs, these sensors are not linear and they present considerable drift and hysteresis [58]. FSR response is usually an inverse power-law, *i.e.*, there is a turn-on threshold, which is a substantial resistance drop at the beginning of the force measurement range. In addition, saturation occurs at the end of the force measurement range. Finally, some authors mention latency and robustness problems in the use of FSR in DMIs [85]. In addition to calibration using curve fitting and a reference measuring system, it is recommended to use conditioning circuits that are able to protect the sensor and the electronics circuits and reduce the measurement error.

#### 4.2.1. FSR Application

Some manufacturers supply a manual [84]. Suggested tips for FSR application are listed below:

- Apply the sensor to a firm, flat and smooth mounting surface;
- Use thin, uniform adhesives;
- Protect the sensor from sharp objects;
- Avoid excessive shear forces;
- Limit the applied current to 1 *mA/cm*^2^ of applied force, as FSRs have a limited power dissipation.

#### 4.2.2. Signal Conditioning

The simplest and most commonly deployed conditioning circuit is the voltage divider. The voltage divider is limited due to a couple of reasons. First, in this circuit, the current applied to the sensor depends on the resistances involved: sensor resistance and series resistor. A careless choice of series resistor values can be dangerous in terms of exceeding the maximum power requirement for the sensor, which can be permanently damaged. Yet another issue is that the voltage output is dependent on the load that it is connected to. This means that load impedance variations affect the measurement directly, unless the load impedance is much higher than the voltage divider resistances. Finally, voltage dividers are vulnerable to noise and are not capable of providing amplification.

**Figure 7.**
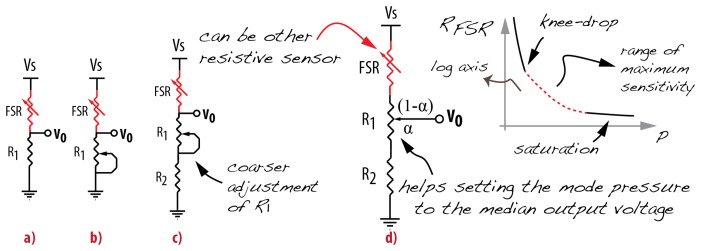
Voltage divider topologies. (**a**) simplest solution; (**b**) output offset adjustment; (**c**) safety improvement; (**d**) safety and finer adjustment.

The simplest way to adjust the output sensitivity of a voltage divider is shown in Figure 7a. This voltage divider does not allow output offset adjustment. In order to allow for this adjustment, the fixed resistor has to be substituted by a potentiometer (Figure 7b). This solution using only two terminals of the potentiometer is troublesome because the current through the FSR depends on not only its own resistance (*R_FSR_*) but also the adjustment of the potentiometer resistance (*R*_1_). As the adjustment of *R*_1_ ranges from its minimum value (0 Ω) to its nominal resistance $R_{1}^{\text{max}}$, the following problems can arise when the potentiometer resistance tends to zero:

- The output can be connected directly to the ground, therefore not measuring the sensor output;
- The maximum current allowance for the sensor can be exceeded, permanently damaging the sensor;
- The power supply might not be capable of providing the demanded power, reducing its voltage and altering the output voltage.

An improvement is the solution presented in Figure 7c. In this case, a fixed resistor is added to the series circuit in order to guarantee the maximum power requirement. The protection considerations determine the value for *R*_2_. Let *V_s_* be the power supply voltage, $I_{A}^{\text{max}}$ the maximum current per area of applied force given by the manufacturer (in *A/cm*^2^), *Area* the area of the sensor's active surface (in *cm*^2^), $R_{\text{FSR}}^{\text{min}}$ the minimum FSR resistance (maximum pressure) and *R*_1_ as the nominal potentiometer resistance. Considering a 10% safety factor, the condition for sensor safety is:

(2) $$R_{\text{FSR}}^{\text{min}}+R_{1}+R_{2}>\frac{V_{s}}{0.9I_{A}^{\text{max}}\text{Area}}$$

Note that in this case, the current through the sensor changes according to its own resistance (*R_FSR_*) and the resistance variation of *R*_1_. In order to overcome this issue, we recommend the circuit in Figure 7d. This is the most efficient manner to use a voltage divider as an FSR conditioning circuit. This topology using the three terminals of the potentiometer provides a finer adjustment of the output sensitivity, which depends on the measurement range, as the sensor response is not linear. Therefore, the *R*_1_ adjustment helps setting the output offset—the minimum voltage output—and consequently the sensitivity level. The designer may avoid operating on the troublesome initial and final measurement range of the FSRs, exploring the range of maximum sensitivity free of saturation.

Practical considerations for resistors selection and adjustment are:

- The ratio *R*_1_/*R*_2_ along with the FSR resistance range defines the measurement range;
- *R*_1_ adjustment defines the offset of the output value;
- Choose *R*_2_ much smaller than *R*_1_, and enough for protecting the sensor (Equation (2));
- Center the output voltage to the most frequent pressure (mode). For that, simulate this pressure and adjust *R*_1_ in ways to obtain half of the supply voltage in the output. If *R*_1_ = *α R*_1_ + (1 − *α*) *R*_1_, this is obtained when $(2\alpha -1)\;R_{1}=R_{\text{FSR}}^{\text{mode}}-R_{2}$.

The optimal choices for *R*_1_ (nominal and adjustment) and *R*_2_, according to the pressure measurement range, can lead to a design whose output measurement range lies within the maximum sensitivity of the sensor, saturating the output during FSR's initial and final measurement range [86]. This improves the curve fitting and takes advantage of the maximum sensor sensitivity.

**Figure 8.**
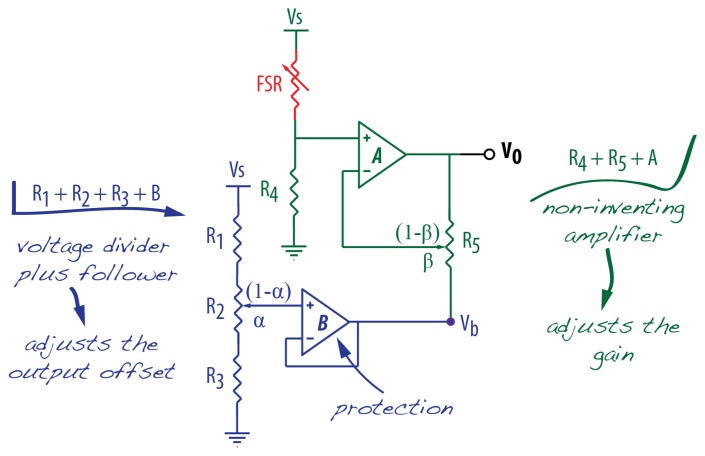
Buffer with adjustable gain and offset.

An improved version of the voltage divider is to have its output applied to a voltage buffer amplifier. This circuit exploits the fact that op-amps have very high input impedance and very low output impedance. The impedance correction protects the sensor from excessive loads coming from the circuit connected to the op-amp output and isolates the output from the high impedance of the voltage divider. In this configuration, the load resistance does not influence the voltage divider output. The op-amp connected as a *buffer* works as a *follower*, that is, the op-amp output voltage follows the op-amp input voltage. The schematic for this circuit is included in Figure 8, which consists of the connection and use of the following components: *R*_1_ as FSR, *R*_2_, *R*_3_ and *B*.

An improved conditioning circuit provides not only output offset adjustment, but also amplification factor (gain) adjustment. The independent adjustment of these two variables can yield a better voltage output range to the given pressure measurement range. For this, an uncomplicated set of op-amps can be deployed, as depicted in Figure 8.

The output for this circuit is:

(3) $$V_{o}=\frac{V_{S}R_{4}}{\beta (R_{4}+R_{\text{FSR}})}-\frac{V_{b}(1-\beta )}{\beta }$$

(4) $$V_{b}=\frac{\left(R_{3}+\alpha R_{2}\right)}{R_{1}+R_{2}+R_{3}}V_{S}$$

where *R*_2_ adjusts the offset, *R*_5_ adjusts the gain, and *α* and *β* are the resistance ratios for the two potentiometers. An important remark is that the offset and gain adjustments *by software* do not have the same role as *by hardware*. If the choices for resistances in the voltage divider result in a short measurement range, an offset adjustment *by software* would not improve the poor sensitivity and/or resolution. Software adjustments are meant for fine adjustments, rather than Signal-to-Noise Ratio (SNR) improvements.

**Figure 9.**
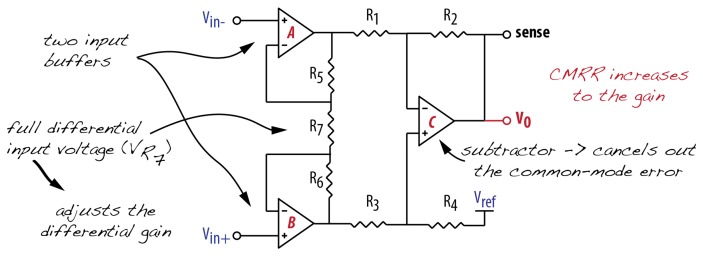
Instrumentation amplifier.

Finally, if the FSR is intended to be used as a quantitative data source, the instrumentation amplifier might be the best option for signal conditioning (Figure 9). This amplifier topology is indicated for improving the SNR of low voltage signals. Its main advantages are very high input impedance, high Common-Mode Rejection (CMR), low DC (Direct Current) offset and low offset voltage drift. The differential amplifier designed using three op-amps is not equivalent to the micro-machined instrumentation amplifier (in-amp) [24]. The reason for that resides in the fact that the differential amplifier must have matched resistors in order to achieve a high CMR—what is difficult to obtain in practice, whereas the in-amps have internal pre-trimmed resistors. The in-amp has a differential input and a single-ended output with respect to a reference [24] and it can be found with dual- and single-supply designs. Normally, the reference pin in single-supply in-amps is set to half power supply level and the output is rail-to-rail [25]. An ideal in-amp detects and amplifies only the difference in voltage between the two inputs, therefore any common-mode signal such as noise or supply voltage drops are rejected.

Alternatively, one might want to detect a force threshold as the sensor is recommended for qualitative purposes. For this, a voltage comparator using an op-amp can be used. The circuit compares the non-inverting input voltage—determined by the voltage divider including the sensor—with the inverting input voltage. The threshold from which the voltage output will switch from low to high is determined by the reference voltage connected to the inverting input. This comparator presents an issue: the output fluctuates in the vicinity of the threshold, especially for slow moving input signals. In order to overcome this drawback, one can use the Schmitt Trigger (ST) comparator. The ST comparator has stable switching between low and high states, which occurs at different levels according to the current output state. The reference inputs depend on the current output state: one for when output is low (*V_l_*), and another for when output is high (*V_h_*).

**Figure 10.**
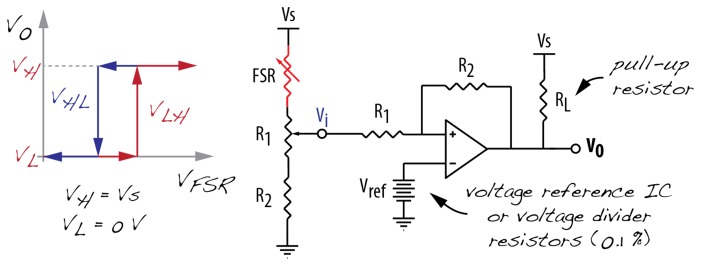
Comparator with hysteresis.

Figure 10 shows the schematics and the response curve of this qualitative conditioning circuit. Figure 10 shows the sensor attached to the inverting input. This way, the output voltage increases with increasing force. Alternatively, this can be easily changed by swapping the sensor and its series resistor. In addition, in order to guarantee complete integration of the output with digital circuits, diodes attached to the output could be used to regulate the voltage levels.

### 4.3. Strain Gage

These reliable sensors are widely used in industry—usually for maintenance and safety—and in research—usually for solid mechanic analysis of materials and mechanical structures. They have also been used in a few DMI applications [14,87]. As the name states, strain gages measure strain caused by stress in a given material. The stress source can be normal, shear, residual, thermal, *etc.* The most common types of strain gages are piezoresistive (*i.e.*, metal and semiconductor) and piezoelectric. The sensitivity—Gage Factor (GF)—is usually 2 for metallic strain gages, and ranges from 80 to 130 for semiconductor strain gages. Our focus is on metallic ones, which sensitivity reflects the ratio between relative resistance variation and strain. The operating principle of a strain gages is based on a clean and thin bond between sensor and surface. This guarantees that the sensor will suffer the same strain as the Device Under Test (DUT).

Metallic strain gages rely on the principle of electrical conductors, whose resistance changes with mechanical stress. The resistance change is due to two factors: the change in the resistivity and the deformation of the conductive material. For an isotropic material, in the linearly elastic region of its response, this is expressed by the following Equation (5):

(5) $$\frac{dR}{R_{0}}=[1+2\nu +C(1-2\nu )]\frac{dl}{l}=GF\frac{dl}{l}=GF∊$$

where *R*_0_ is the resistance without any stress, *ν* is the Poisson ratio, *C* is the Bridgman constant, *GF* is the sensitivity and *∊* is the strain. Both *ν* and *C* are intrinsic to the material: *ν* is the transverse to axial ratio whereas *C* is the ratio between resistivity variation and volume variation. Strain and consequently resistance variation are very small, which makes strain hard to be measured. It is important to note that the absolute value of the resistance does not carry information about the strain. It is indeed the resistance variation the variable to be analyzed.

Balance conditioning circuits like the Wheatstone bridge can be deployed to measure small resistance variation [88]. The bridge is intended for common-mode rejection, in this case, rejecting the common nominal resistance of two resistances and/or strain gages. The bridge loses its balance when the sensor resistance varies due to a strain. For low SNR bridge output voltage, specialized conditioning solutions are required, which are briefly reviewed in Section 4.3.4.

#### 4.3.1. Temperature Effect

Temperature is the main interference quantity when using strain gages. Temperature and its variation have effects on DUT and sensors. Given a perfect bond between sensor and DUT, the strain on the DUT is transferred to the strain gage.

The main effect of temperature variation on the DUT is the change of its dimensions (visible or not). This occurs because of the *thermal expansion coefficient* of the DUT's material. The thermal longitudinal expansion has an effect on the strain measurement. It is impossible to distinguish the cause of strain: mechanical or thermal. One solution for that is in the sensor manufacturing. Some sensors are designed in such a way to have the same thermal expansion coefficient of the material that they are intended to be applied to. This way, both the DUT and the sensors are going to change their dimensions at the same rate, partially compensating for thermal stress in the sensor. Strain gages endowed with thermal treatment are called *strain gages with matched temperature coefficient* [22]. In order to take advantage of this method, the DUT's material and the sensor should have similar thermal coefficients.

Another source of thermal error while measuring strain occurs in the sensor itself. The sensor grid also changes its dimension in accordance with temperature changes. This effect can be compensated for using the balance principle of a Wheatstone bridge and an additional sensor. The second sensor, identical to the first one, is not submitted to mechanical strain and should be installed in the same material as the DUT and in such a way be exposed to the same temperature. This second sensor, submitted to thermal strain only, is then properly placed in the Wheatstone bridge in order to subtract the thermal effect in the overall voltage output [89].

Although these are the main effects of temperature on strain measurement, temperature of lead wires and power dissipation in the sensor can also be sources of artifacts.

#### 4.3.2. Selecting Strain Gages

The selection of a strain gage for a certain application is one of the most important tasks in the whole procedure. The designer should take into account the material of the DUT, the type of stress to which the DUT will be submitted (axial, shear, residual, *etc.*), the expected strain magnitude, the stress state (uniaxial, biaxial or triaxial) and the possible sources of artifacts.

#### 4.3.3. Strain Gage Application

The application of strain gages to a DUT is a delicate and complex procedure. Special materials and a rigid protocol should be in place in order to guarantee a perfect bond between sensor and DUT. Yet another layer of complexity resides in sensor protection, since they are delicate.

#### 4.3.4. Signal Conditioning

A signal conditioning circuit for strain gages includes the application of the following conditioning technique and circuits: Wheatstone bridge configuration according to the stress profile, temperature compensation, zeroing circuit, lead wire compensation, amplification (the instrumentation amplifier shown in Figure 9 is an option) and filtering (if necessary).

Following that, a calibration using a reference measuring system should be used. A bullet-proof protocol for strain gage measurement and data analysis is given by the *Unified Approach to the Engineering of Measurement System for Test and Evaluation* [2]. This method synthesizes calibration, control, test and evaluation of measuring systems. One of its main contributions is the discussion of interference quantities and tests to detect their influences.

## 5. Sensing Recommendations for DMIs

In this section, we aim to offer suggestions for DMI designers. As mentioned earlier, the use of specialized sensors and conditioning circuits, as well as the use of sensor fusion, can improve not only the accuracy of DMIs but also the user experience. Previous work has shown that the use of specialized sensors can be a significant determinant of classifying musical gestures, allowing for different mapping according to the gesture being performed [42]. In this particular case, the strain gages in the DMI (The Rulers) were capable of determining the instants when the user was interfering in the inertia of the instrument. That is, using an analogy of a plucked string, the strain gages have a different response—transfer function—during intervals where the user is acting upon the string by displacing it, and during intervals where the string is vibrating according to its own physical properties [42]. This quality of the strain gages cannot be found in any of the other ordinary sensors previously used for the same instrument: infrared and Hall effector sensors [90].

### 5.1. Use of Specialized Sensor Technologies

In order to suggest improvements, we have selected some DMI examples and will review their reported measuring techniques.

#### 5.1.1. Measuring Trumpet Valve Position

The challenge of measuring the valve position on a trumpet was approached with the use of potentiometers and buttons [91], and later by digital infrared proximity sensors [92]. The clarity of the latter paper allowed for the identification of the sensor as a VCNL4000 [93]. According to this sensor's datasheet, the infrared sensor is not linear and does not have a monotonic response. Nonlinear sensors require high order fitting curves that can be computationally expensive for ubiquitous microcontrollers. In addition, nonlinear responses present variation in sensitivity along the measurement range that must be accounted for while calibrating the system. The worst possible choice would be to fit the infrared response to a linear curve, yielding to errors along most of the measurement range [90]. A non-monotonic response occurs when a sensor presents the same output in response to multiple different inputs (proximity, in this case). A partial solution for this ambiguity would be to evaluate the previous measurement in order to obtain the instantaneous derivative of the signal. The sign of the derivative would partially solve the singularity issue. However, the sensitivity closer to the point of zero-derivative tends to zero, which is not at all desirable. Finally, the solution for nonlinear, non-monotonic sensor responses starts with good placement, calibration and coherent curve fitting (polynomial order higher or equal to 2) [90]. Another common issue using infrared sensors is saturation. The remedies described above can account for that as well.

Some developers mentioned that the sensor has a high sensitivity and that its placement is a critical concern [92]. Then, they built a series of tests to find an optimal placement that would provide the highest linearity, highest dynamic (measurement) range and robustness. These efforts are a good example of engineering development and evaluation.

Some suggestions could further help designers in selecting the best placement. One of them would be to evaluate monotonicity, linearity, saturation, sensitivity and dynamic measurement range along the valve operation range. Another recommendation would be to perform a calibration where the measurements provided by the sensors are compared with a reference. Calibration implies comparison with a truth value [1] and should not be misinterpreted as a measurement range adjustment. Therefore, the concept of “self-calibration” is flawed. In terms of linearization, if higher polynomial orders cannot be applied due to processing capability, a lookup table or conditional multiple linear measurement range can be alternatives [86].

Yet another possibility for this case would be the use of linear specialized sensors. Thibodeau and Wanderley have compared slide potentiometers, Hall effect sensors, non-infrared LED to LVDT—Linear Variable Differential Transformer—sensors, for the specific issue of measuring position of trumpet valves [56]. The comparison of sensor responses was done through calibration along the valve operation range. LVDT sensors are linear and monotonic; therefore, all issues presented by the use of infrared sensors are eliminated [52].

#### 5.1.2. Measuring Key Pressing on a Piano or Gamelan Instrument

A lot of NIME papers could make use of strain gages, especially those that aim to measure stress on metallic/plastic parts of piano or gamelan instruments [85,94–97]. The most used sensors in these references are infrared and FSR sensors. One example studied the actuation and sensing of electromagnetically sustained piano [95,98]. This interesting work makes use of piezoelectric discs and infrared sensors. The infrared sensors measure the deflection of the cantilever beam. This issue is solved by the use of strain gages in other works [42,90]. A remark must be made about the essential use of protective buffers for piezoelectric discs, as these can deliver voltages that might not be supported by the conditioning circuits or microcontrollers. Another example using infrared sensors improves the output response through the use of coherent conditioning circuits [94].

As cited before, infrared sensors are nonlinear and non-monotonic. In addition to that, for a cantilever application, there are two other issues that can compromise the measurement quality. The first one is the deflection angle of the cantilever beam. Infrared sensors usually work by providing an infrared beam and reading the respective infrared reflection. The reflection reading depends on the flatness of the reflective surface, guaranteeing the symmetry of incident and reflective light beams. If the flatness cannot be guaranteed due to the angular cantilever deflection, errors might occur as this angle increases. The second issue comes from the use of shiny cantilever beams. Some coating surfaces can interfere with the infrared reflection.

The examples based on FSR might suffer from artifacts related to flatness of the surface, mechanical robustness, latency, nonlinear response and deficient conditioning circuitry [85,97]. For these examples, we recommend the use of strain gages and sensor fusion techniques. The strain gages' main advantages for this particular application are linearity, high SNR (provided by coherent conditioning circuits), and no electromagnetic interference. Finally, strain gages could potentially distinguish between touch and after-touch intervals, serving as a reference for the adaptive gain desired by the instrument designers. Furthermore, sensor fusion techniques could be deployed to improve sensing and control. Based on our previous work, instrument designers could take advantage of the known physical model of the cantilever beam and also eliminate high frequency harmonic vibration responses by limiting the physical model of the cantilever to a low order system [42].

#### 5.1.3. Measurements on Violin and Cello: Distance, Force, Position, Fingering

Some interesting studies in NIME are dedicated to measuring hair deflection for tracking and force assessment [99,100]. One of them presents an interesting triangulation method to track the bow, using infrared sensors [99]. The other work is based on tracking using Polhemus^©^ motion capture [100]. The force information is a result of calibration using a load cell with applied strain gages [101]. The drawback of this approach is the magnetic artifacts on the tracking data. Yet another work on string instruments uses FSRs to measure finger position and pressure [97]. The main artifact of the chosen design is the application of the sensor in a curved surface [97,102], as the contraction and extension created by a curved surface can be interpreted as pressure by the sensor. Another issue is the conditioning circuit. The author's design is a voltage divider followed by a zero-gain amplifier (buffer). This voltage divider configuration can lead to a short circuit in the power supply when a critical, however possible, situation occurs: high values of pressure on the sensor while the potentiometer set to a low resistance. According to Section 4.2.2, we recommend the circuit in Figure 7d as voltage divider for FSR applications.

In contrast to artifacts in infrared sensors, FSRs and magnetic tracking systems, a solution using fiber optics proved to be successful for measuring fingering, bow speed, and bow pressure in an fMRI-compatible cello [103,104]. This is another example of how the use of specialized sensor and sensor conditioning circuits are superior in terms of reproducibility and accuracy, and offer further possibilities to a DMI designer. For instance, the complexity of the conditioning circuit required the design of an optoelectronic acquisition and control board [105]. A more simple but effective solution is the use of capacitive sensing for bow pressure and position [106]. Yet another alternative for measuring pressure on the fingerboard is the use of paper-based force sensors or printed capacitive sensing [54,55,107].

### 5.2. Sensor Fusion

From the review presented in Section 2, forty percent of the NIME publications in the recent years use more than one sensing technology. This considerable percentage allows for sensor fusion application. In this section, we briefly describe the sensor fusion domain and introduce two algorithms: complementary filtering and Kalman filter. Firstly, it is interesting to note the difference between *multisensor integration* and *sensor fusion*. The former means the synergistic use of multiple sensor data directly processed by the control application, that is, a direct mapping topology for multivariable systems. The latter uses multiple sensor data to generate another layer of sensor data that will then be mapped to a control process [108]. A common concept among several authors is that the product of a sensor fusion implementation is an information set that is better than the information gleaned from individual sources [29,108–111]. Some add that it provides a statistical improvement proportional to *N*^1/2^, where *N* is the number of independent observations [29]. The human being's capacity to fuse information in order to convey meaning is often used as a reference for the information fusion community. Technology systems try to imitate this faculty as well as its connection to decision making. Common limitations in most measurement systems justify the use of sensor fusion techniques. Some of them are [108,109]:

**Sensor deprivation:** breakdown of a sensor, causing information loss at a range, device or quantity;
**Limited spatial coverage:** range limitation of sensors, such as measurement scale and placement position;
**Limited temporal coverage:** the required time to perform a measurement and a transmission operation, thereby defining the sampling frequency;
**Metrology limitations:** metrology characteristics of sensors, such as resolution and errors;
**Susceptibility to the environment:** susceptibility of the system to interference that may degrade performance.

Techniques for sensor fusion make use of observations made by sensors to estimate a process state vector. Fusion techniques are usually used for smoothing, filtering and/or predicting. Some potential advantages of sensor fusion systems are:

**Redundancy:** the property of providing information even in case of partial fault or data loss from one or multiple sensors;
**Enhanced spatial or geometrical coverage:** overall better coverage obtained by using complementary sensors;
**Enhanced confidence:** measurements of one sensor confirm data from another sensor, hence overall statistical indicators may experience improvement;
**Ambiguity reduction:** controversial measurement scenarios can be solved by analyzing multi-sensor data;
**Enhanced robustness:** different sensors can present diverse robustness against a significant interference quantity Fusing data in a manner that at least one sensor is robust against that interference quantity improves the overall robustness of the system.

However, sensor fusion responses also present limitation such as:

**Requirement of good measurement sources:** there is no general improvement based on bad input data. Additionally, sensor fusion techniques applied over bad input data might reduce the overall performance by introducing time delays or unwarranted confidence [112];
**High processing and communication power:** the implementation of sensor fusion procedures in embedded and real-time applications can be unviable or limited;
**Tools' selection:** the selection of inappropriate techniques for a certain problem might degrade the original performance;
**Requirement of process model knowledge:** some sensor fusion algorithms, such as the linear Kalman filter, strongly rely on the knowledge of the system process model description. An inaccurate physical modeling of the system and its measurements can lead to instability and inaccuracy [31].

The section below presents a brief review on complementary and Kalman filter, two of the most commonly used techniques [27,113].

#### 5.2.1. Complementary Filter

Complementary filter is a filter topology that can be applied alone or as a preprocessing technique for Extended Kalman Filters (EKF), for instance [41]. It is reasonable to explain its implementation by using frequency domain formulations. As an example, one can assume a system based on two measurement devices whose outputs are *y*_1_(*t*) and *y*_2_(*t*) and are represented by:

(6) $$y_{1}(t)=s(t)+n_{1}(t)$$

(7) $$y_{2}(t)=s(t)+n_{2}(t)$$

where *n*_1_ and *n*_2_ are measurement noises and *s* is the unknown signal to be determined. The measured signals are considered stationary random process with known power spectral densities. The signal *s*(*t*) is unknown and can be deterministic or non-stationary The aim of a filter is to optimally produce an estimate *ŝ*(*t*) of the unknown signal *s*(*t*), assigning weights for the noisy measurements such as described in the following equations:

(8) $$ŝ(t)=G_{1}(s)y_{1}(t)+G_{2}(s)y_{2}(t)$$

(9) $$Ŝ(s)=(G_{1}(s)+G_{2}(s))S(s)+G_{1}(s)N_{1}(s)+G_{2}(s)N_{2}(s)$$

Equations (8) and (9) are respectively the time and frequency representations for the complementary filter. In order to avoid distortion, the following constraint must hold for all *s*(*t*):

(10) $$G_{1}(s)+G_{2}(s)=1$$

The complementary filter is consider to be a “safe” solution, as it is particularly suggested for cases where the filter might need to cope with statistically unusual situations without resulting in large errors [28]. Complementary filters are usually applied for systems containing sensors with complementary spectral characteristics, as it is the classical example of inertial sensors.

#### 5.2.2. Kalman Filter

Essentially, the Kalman filter combines different measurement outputs in a systematic and optimal manner, given the prior knowledge about the system and measuring functions. The filter's goal is to provide an estimate of desired quantities, whose errors are improved in comparison with the original data sources. By carrying this out, Kalman filters can minimize the estimated error covariance and compensate for limitations of the sensing system such as lag and drift.

Some authors argue that the average results of the Kalman filter are better than the average results of any other filters used for sensor fusion [27,114]. The filter has been widely used for tracking purposes [31], motion prediction and orientation estimate (mostly with MARG sensors) [27,115,116], navigation and process control [117]. Additionally, Kalman filters have a good real-time and online application potential [109]. They are said to be optimal as they use the maximum number of statistical descriptors to evaluate a system-measurement problem. Comparatively, other filter topologies provide approximated results for the estimate covariance, since they do not require complete and accurate knowledge about the system, measurements and their statistical descriptors.

The description of system, measurements and their statistical descriptors is not always possible or fully definable. For these cases, other filter topologies such as particle filters can be deployed [118,119].

The linear version of the Kalman filter is the one that provides the best results, with the cost of restricted requirements: zero-mean Gaussian noises and linear stochastic models for system and measurements. Based on the levels of confidence on measurements and system, the Kalman filter delivers the estimates of the system variables and the covariance of those estimates. In a successful linear Kalman filter implementation, the covariance of the estimate is smaller than any of the elements of the measurement covariance matrix main diagonal.

However, the application of these techniques to gesture analysis is not straightforward as there is no clear physical model describing the gestures used to control DMIs. Some research circumvents this problem by setting an enclosed gesture vocabulary for which the physical models can be accurately defined [33,34]. This approach is not reasonable for musical gestures as it would excessively limit playing techniques.

Therefore, regarding skilled motor performance, it is impossible to define rules, patterns, probability or sequences for the human input that could well describe the process model of the system. In order to overcome that, previous work has developed a framework to apply sensor fusion to DMIs and other unpredictable signal devices [42]. The solution was based on a multiple-model linear Kalman filter in combination with gesture/motion segmentation. The motion segmentation discriminates gestures according to the knowledge of their process model. This allows a more predictive estimation during periods of free motion, while relying on a less predictive approach for unknown user-driven signals [42]. Results reveal that the proposed method improves the error covariance of the estimate by a factor of 2.2 for driven motions and 12.7 for free motions in comparison with single-sensor filter design.

The structure for this framework is presented as follows [42]:

- Offline tasks are performed in a heuristic manner:
  **Motion segmentation:** identification of distinct gestures with distinguishable physical model or measuring models;
  **Regression:** different slopes and intersections regression for each of the identified gestures;
  **Evaluation:** evaluation of optimal physical modeling for processes and measurement functions, for each identified gesture.
- Online tasks performed for each upcoming sample:
  **Data acquisition:** synchronization and alignment of multiple sensor data must be guaranteed;
  **Classifier:** classification of the gesture being performed and activation of the corresponding filter;
  **Measurement function:** computation of measurement output using the corresponding regression for the gesture being performed;
  **Kalman filter loop:** use of physical modeling, system and measurement error covariance matrices correspondent to the gesture being performed.

#### 5.2.3. Sensor Fusion for MARG Sensing

Interesting examples of sensor fusion for human application using MARG sensing range from complementary filters to Kalman filter [33,46,120]. Complementary filtering fuses accelerometer and magnetometer data through the use of low- and high-pass filters, whereas the Kalman filter can potentially correct for noises in the yaw angle [41]. A framework for the application of Kalman filters for orientation purposes using MARG sensors is introduced as follows:

- Ensure alignment of all MARG sensors in case they are not packed together;
- Calibrate inertial sensors;
- Calibrate magnetometers and compensate for soft- and hard-iron artifacts;
- Obtain systematic and random errors of all sensors. This information is necessary for defining the measurement covariance matrix in the Kalman filter;
- Know your process: design the physical model of your process. For human input processes, refer to [42];
- Apply the Kalman set of equations.

## 6. Conclusions

We have presented an overview of sensor use manifested in the NIME Conference proceedings from 2009 to 2013. The survey included total sensor use, use trends, use of motion capture tools and portable consumer electronic devices, concomitant use of sensors within the same application and embedded use of MARG sensors in portable consumer electronic devices. Then, we presented a brief overview on human motion analysis, discussing its kinematic and kinetic measuring methods. We showed that the most used sensors in DMIs provide force-related variables: accelerometer and FSR.

We then presented an overview of accelerometer and FSR use concerning techniques of conditioning circuit and signal processing. We pointed some limitations of recurrent designer choices, offering alternatives for a more advanced instrumentation design. We also proposed as alternative for force acquisition the use of strain gages, a progressive and versatile choice with linear and reproducible response.

Then, we commented on interesting examples of sensing application in NIME publications, once again proposing improvements for the instrumentation design and sensor signal processing through the use of *specialized sensors, advanced electronic conditioning circuits* and *advanced sensor signal processing*. Regarding the advanced sensor signal processing, we focused on sensor fusion. We remarked that for human input signal, as it is the case for DMIs, most of the sensor fusion techniques are not straightforward, because human input signals are not trivially modeled. Therefore, despite the improvements brought by sensor fusion techniques, this method alone is not sufficient to obtain accurate signals.

Overall, we concluded that there is an urgent need to improve instrumentation techniques for DMIs and other human input devices, in order to design reliable instruments that are also robust, reproducible, responsive and accurate. We believe that efforts on improving sensing design—through the use of state-of-the-art engineering techniques—on DMIs can bring improvements concerning explorability and feature controllability [12]. Finally, as shown by the review of sensor use, there is an expansion in the use of MARG sensors for motion analysis. The current challenge is to fuse kinetics and kinematics for better analyzing human motion. Our future work is devoted to algorithms for biomechanical analyses that fuse positional data with sensor data—especially MARG sensors.

## Figures and Tables

**Table 1.** Dataset of sensor use in NIME conference proceedings from 2009 to 2013, compared with respective dataset of previous study [10]. MARG sensors have two occurrence values: the total number, and the embedded number of occurrences (in parentheses).

**Sensors**	**Occurrence (2009–2013)**	**Occurrence (2001–2008) [10]**
accelerometer	75 (30)	56
FSR™ (Force Sensing Resistors™) ^1^	38	68
buttons and potentiometers ^2^	29	110
gyroscope	30 (9)	
video/image ^3^	23	54
IR (infrared) ^4^	22	27
magnetometer	16 (4)	
capacitive	15	
biosensing ^5^	13	
piezoelectric disc	12	
non-definable ^6^	12	
microphone	11	29
Textiles	11 ^7^	
photo/light	10	
Bend	9	21
Hall effect	7	
ultrasound	4	
pressure/flow	4 ^8^	
fiber optic	2	

1 *FSR* and Force Sensing Resistors are trademark of Interlink Electronics. In this text, we adopt the community's understanding of these terms: resistive sensors for measuring pressure. Therefore, we excluded the ™ symbol to refer to any alike sensor, disregarding the brand [55,58];

2 This work combined *all potentiometers and switches* used as sensors within one category, whereas the previous work classified these sensors as button and switches (51 occurrences), rotary potentiometers (31 occurrences) and linear potentiometers (28 occurrences) [10];

3 *video/image* category does not include video from Kinect^©^;

4 *infrared* category does not include the infrared sensing embedded in the Wii^©^;

5 the *biosensing* category refers to all biosignal sensing: EMG (ElectroMyoGraphy), EEG (ElectroEncephalography), *etc.*;

6 the *non-definable* category includes the instances where neither the sensor used nor the quantity being measured were possible to determine;

7 *textiles* were mostly used as resistive sensors;

8 *pressure/flow* category relates to airflow measurements.

**Table 2.** Average use per year, compared with previous study [10].

**Sensors**	**Average Use per Year (2009–2013)**	**Average Use per Year (2001–2008) [10]**
accelerometer (embedded or not)	15	7
FSR	7.6	8.5
buttons and potentiometers (all)	5.8	13.8
video/image	4.6	6.75
infrared	4.4	3.4
microphone	2.2	3.6
bend	1.8	2.6

**Table 3.** Non-exclusive occurrence by class.

**Occurrence by Class**		
sensors	analog	172
	digital	134
others	consumer electronics	71 ^1^
	motion capture	30

1 at least one type of MARG sensor is used in 43 occurrences.

**Table 4.** Co-occurrence matrix of MARG sensors in portable consumer electronic devices.

**MARG Sensors**	**Devices**	

	**Wii^©^**	**Cell Phones/Tablets/Music Players**
accelerometer	7	24
gyroscope	1 (MotionPlus^©^)	8
magnetometer	0	4
